# Minimal phenotypic test for simple differentiation of *Xanthomonas campestris* from other yellow-pigmented bacteria isolated from soil

**Published:** 2011-06

**Authors:** MR Soudi, N Alimadadi, P Ghadam

**Affiliations:** 1 National Laboratory of Industrial Microbiology, Department of Biology, Faculty of Sciences, Alzahra University, Vanak, Tehran, Iran; 2 Department of Biology, Faculty of Sciences, Alzahra University, Vanak, Tehran, Iran

**Keywords:** biochemical tests, soil, xanthan, *Xanthomonas campestris*, yellow-pigmented bacteria

## Abstract

**Background and Objectives:**

Isolation of *Xanthomonas campestris* from soil has a wide range of applications from monitoring of phytopathogenic populations in soil to screening of improved xanthan-producing strains. Identification of *Xanthomonas campestris* and its pathovars requires pathogenicity tests in addition to phenotypic and molecular characterization.

**Materials and Methods:**

Thirty phenotypic tests were carried out on 57 yellow-pigmented bacterial isolates obtained from soil of cabbage farms after screening on Selective Xanthomonas (SX) agar and transferring on Yeast Malt agar. Absorption spectra of pigments and capability of biopolymer production were determined for the isolates. Some characteristics of the biopolymer produced and presence of a *X. campestris*-specific gene marker were investigated for nine putative *X. campestris* isolates.

**Results:**

The present study introduces a set of simple phenotypic tests including urease, acid production from sucrose, mucoid growth on 5% sucrose, starch hydrolysis, growth in 4% NaCl, motility and utilization of asparagine as sole carbon and nitrogen source for quick and inexpensive tentative identification of *Xanthomonas campestris*. Validation of these tests was confirmed in 100% of the cases by characterization of bacterial exopolysaccharide as xanthan and production of genus-specific xanthomonadin pigment. Moreover, tracking of *hrc* gene among putative *X. campestris* isolates gave positive results in 80% of cases.

**Conclusion:**

The Minimal simple phenotypic tests facilitate the screening and differentiation of putative *X. campestris* isolates from other false bacterial strains isolated from soil on semiselective SX agar.

## INTRODUCTION

Xanthan gum is a microbial exopolysaccharide produced by the phytopathogenic bacterium *Xanthomonas campestris*. It is widely used as a food ingredient and as one of the main components in water-based drilling fluids in petroleum industries. In addition, due to its unique rheological and other physicochemical properties, it plays various roles in a broad range of industries such as toiletries, cosmetics, water-based paints, textile, ceramics, etc. (1). The global production of xanthan by commercial providers exceeds 86,000 tons annually (2) and the demand for xanthan gum is estimated to grow continuously at an annual rate of 5–10% (1).

While nearly all *Xanthomonas* spp., specially *Xanthomonas campestris* pv. *Campestris*, may produce xanthan gum (3), most studies report a clear dependency between strain used and xanthan yield and properties (4, 5). Thus, isolation and identification of new strains of *X. campestris* can be a continuous struggle for providing the opportunity of increased yield of xanthan gum and achieving increased rheological quality products (6–10).

*Xanthomonas* species, all phytopathogenic bacteria, belong to *Xanthomonadaceae*, the sole family in the *Xanthomonadales* order, in the γ-Proteobacteria. Xanthomonads, as with other plant pathogens, may be transmitted to a new host, and particularly in this case, a period of survival in the absence of the host is necessary. Such survival may be achieved in many ways, such as with seed, plant residues, and perennial hosts epiphytically or saprophytically existing in soil or on insects (11). Plant debris, seed, and weeds are most often reported to be important sources of inocula for black rot, the disease caused by *X. campestris* pv. *campestris* (12–14). The infection can also occur from infested soil (15, 16). *X. campestris* pv. *campestris*, i.e. the main xanthan producer, thrives especially well in warm and humid climates and survives from season to season in infected seed and even longer in plant debris in soil and it readily spreads to nearby plants by rain splash (17, 18). Although the bacterium primarily is seed-borne, when protected by host debris, it may survive in soil for up to two years (16). The ability to produce exopolysaccharide has been shown to paly an important role in survival of *X. campestris* in soil environments (15).

Identification of Xanthomonas species is usually accompanied by certain difficulties. *X. campestris* currently includes a number of pathovars that cause diseases predominantly within the family *Brassicaceae*. In general, the pathovars are not distinguishable by phenotypic characterization, and identification is reliant on knowledge of their hosts (11). However, the differentiation according to pathogenicity test is often difficult (19) and sometimes these pathovars develop lesions indistinguishable from another (20). Taxonomic studies have not been completed and problems are still faced in naming pathovars of *X*. *campestris* (19, 21–23). PCR protocols for rapid and specific identification of *X. campestris* using hypersensivity response and pathogenicity (*hrp*) genes from *X. campestris* as the molecular targets are available (24–26).

In some areas, such as certain agricultural regions found in Iran with low incidence of diseases caused by *Xanthomonas campestris*, soil of crucifer farms are a good source of *X. campestris* strains with potential in xanthan production industry. A number of other bacteria with morphology similar to *Xanthomonas* spp. are usually isolated during screening procedures from soil. The goal of this study was to establish a series of minimal biochemical tests as a requirement for simple and rapid differentiation of xanthan-producing strains of *X. campestris* from other yellow-pigmented bacteria existing in soil in order to facilitate the screening procedures.

## MATERIALS AND METHODS

**Microorganisms.** Seventy-eight bacterial isolates were obtained from soil of nine cabbage farms in Tehran Province, Iran. The isolates were selected using dual screening steps: first, isolation on semi-selective SX agar medium (27) and then purification on Yeast Malt agar (YM agar) medium (28) for obtaining yellow and mucoid colonies. *Xanthomonas campestris* strain b82, previously isolated from soil in Iran (29), and *X. campestris* DSM 1706 were used as control. Pure cultures of the bacteria were maintained on YM agar slants at 4°C and transferred every 14 days to prevent strains from losing their production capability (30). Long-term preservation was achieved by deep-freezing in Nutrient broth (NB) plus 15% (v/v) glycerin (22, 23) and freeze drying (30).

**Absorption spectra of pigments**. Pigments from all isolates and control strains were extracted by scraping the bacterial cells from the surface of 48 h Nutrient agar (NA) plates and suspending the cells in 3 ml methanol in tightly capped tubes. Each tube was immersed in boiling water bath for 5 min and then centrifuged at 8000 × g for 15 min. The supernatant was allowed to evaporate in a water bath at 50–60°C until the optical density of the extract reached 0.4 at 443 nm (31). The absorption spectrum of the extract was determined using a scanning spectrophotometer (CECIL CE 9050).

**Biopolymer production**. An overnight culture of each isolate was prepared as inoculum by transferring a loopful from a 24-h slant to 5 ml of YM broth and incubating at 28°C. One hundred milliliter Erlenmeyer flasks containing 20 ml of YM broth were inoculated and incubated at 28°C on an orbital shaker at 140 rpm for 8 h. These cultures were used to inoculate 250 ml flasks containing 50 ml of production medium (gl^−1^;sucrose 30, citric acid 2.1, (NH_4_)_2_SO_4_ 1.2, KH_2_PO_4_ 5, and MgSO_4_.7H_2_O 0.24 in tap water; pH 7). After incubation at 28°C and 140 rpm for 72 h, apparent viscosity of fermentation broth was measured at room temperature using a Brookfield system viscometer (Anton Paar, DV1) with spindle number 3 at 60 rpm. Raw product was recovered by precipitation with 1.5 volume of isopropyl alcohol in the presence of NaCl (0.5 gl^−1^) and drying in an oven. The results were recorded as average values of four similar experiments.

For nine putative *Xanthomonas campestris* isolates as representatives, amounts of biopolymer and biomass in the raw product were estimated by heat treatment of reconstituted raw product followed by alkaline protease treatment (1000 Ug^−1^ biopolymer) and then recovered and dried as previously described (32). After partial purification of the products, viscosity of 1% polymer solutions with 1% KCl were measured at 12, 20, 30, 50, 60, and 100 rpm as describe above. The experiments were carried out in duplicate. All the results were analyzed statistically by one-way analysis of variance and Tukey test with 95% confidence level using Minitab (15.2) software.

**Phenotypic features. **Thirty phenotypic features were tested for 57 isolates including 30 putative *Xanthomonas campestris* and 27 randomly selected yellow-pigmented false isolates, as well as control strains. In addition to Gram staining, KOH reaction and motility test, the biochemical and physiological tests were performed including: catalase, oxidase, oxidation-fermentation of glucose, indole and acetoin production, hydrolysis of gelatin and Tween 80, salt tolerance (33), nitrate reduction, H_2_S production, urease and arginine dihydrolase activity, production of fluorescent pigment on King B medium, mucoid growth on NA plus 5% glucose and sucrose, growth at different temperatures and in the presence of 0.1% 2,3,5-triphenyl tetrazolium chloride (TTC), hydrolysis of esculin, starch, and casein, acid production from glucose and sucrose, utilization of citrate and propionate (31), growth at 4°C and pH 4.5 (34), and growth on asparagine medium (35). Pure and fresh (24–48 h) cultures were used to perform all the tests in triplicate.

Identification based on a species-specific gene. For nine putative *Xanthomonas campestris* isolates as the representative isolates and *X. campestris* DSM 1706 as control, the presence of a species-specific gene, *hrcC*, was determined. Total genomic

DNA was extracted from a mass of bacterial cells grown overnight in NB medium, using the phenol-chloroform technique described by Gomes et al. (36) with some modifications. DNA extracts were stored at −20°C and their quality was checked by visual inspection on a 0.8% agarose gel. Amplification of a segment of *hrcC* gene was carried out using primers HrcCF2 and HrcCR2, described by Zaccardelli et al. (26). PCR was performed in a total volume of 25 µl using 1.25 µl DNA, 200 µM dNTPs, 0.5 µM of each primer, 1X Taq buffer, 2 mM MgCl_2_, and1.25 U Taq polymerase. The amplification program consisted of an initial denaturation at 93°C for 3 min, 35 cycles of 93°C for 45 s, 64°C for 30 s and 72°C for 90 s, followed by final extension at 72°C for 7 min. DNA extract of *X. campestris* DSM 1706 was used as positive control and samples lacking template DNA and/or one of the primers were used as negative controls. The amplicons were analyzed by agarose gel electrophoresis and subsequent staining with ethidium bromide. PCR for each isolate was carried out in duplicate.

## RESULTS

**Pigment and biopolymer production**. In this study, 57 isolates were selected on SX medium and purified as mucoid yellow pigmented bacteria on YM agar. Presence of xanthomonadin pigment and capability of xanthan production are unique characteristics of the genus *Xanthomonas* (11). Absorption spectra of pigments extracted from the 30 isolates were similar to that of control strains known as *X. campestris* pv. *campestris*, and absorption maxima were determined in the range of 441.0-444.7 nm. These isolates also produced highly viscous biopolymer in the sucrose-containing production medium (Table 1). Based on these results, the other 27 yellow-pigmented isolates showed absorption maxima between 414.6–425.4 nm wavelengths without production of any remarkable viscosity called false isolates.

**Table 1:** Maximum absorption spectra of pigment extracts and capability of biopolymer production.

Bacteria	λ_max_*of pigment (nm)	Apparent viscosity of culture broth (cP)	Raw product (gl^−^ 1)
Putative *X. campestris*	441.0–444.7	1200	14.41
False isolates	414.6–425.4	1∼	–
*X. campestris* b82	˄,442	1010	12.74
*X. campestris* DSM 1706	442.3	1198	13.24

* Wavelengths in which methanol extract of pigments showed maximum absorption spectra.

**Fig. 1:**
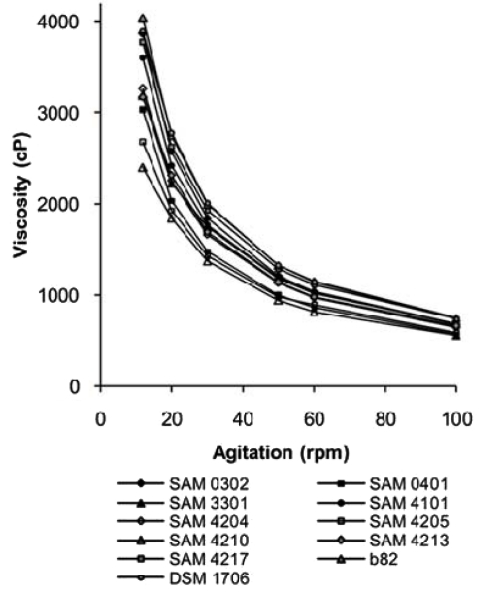
Viscosity of 1% polymeric solutions of xanthan extracted from the representatives of putative *Xanthomonas campestris* isolates and the control strains at different agitation rates.

After partial purification of the raw products, amount of biopolymer and biomass in the product and apparent viscosity of the biopolymer solution was determined for a randomly selected group of nine putative *X. campestris* isolates as the representative of all polymer-producing isolates (Table 2). All polymeric materials extracted from fermentation broth showed typical rheological properties of xanthan gum such as pseudoplastic behavior (Fig. 1), increased viscosity of about 1000 cP in 1% aqueous solution (Table 2) and typical interaction of xanthan with organic solvents. Average amounts of biopolymer and biomass production in the isolates were 9.80 and 3.95 gl^−1^, respectively. Measurement of apparent viscosity of 1% polymeric solutions in 1% KCl resulted in an average of 978 cP using spindle 3 at 60 rpm in Brookfield viscometeric system. No significant difference was observed among parameters studied in the isolates and those of the control strains.

**Table 2:** Amounts of biopolymer, biomass and viscosity in putative isolates of *Xanthomonas campestris*.

Bacteria	Xanthan (gl ^−1^ )	Biomass (gl^− ^ 1)	Viscosity* (cP)
SAM 0302	9.02	4.28	1042
SAM 0401	9.58	4.81	869
SAM 3301	9.32	3.95	1022
SAM 4101	10.73	4.19	1042
SAM 4204	10.22	3.80	974
SAM 4205	7.81	4.05	894
SAM 4210	10.08	3.54	823
SAM 4213	11.21	2.98	986
SAM 4217	10.20	3.94	1120

*X. campestris* b82	9.58	3.17	1153
*X. campestris* DSM 1706	9.52	3.71	1140

* Viscosity of 1% polymeric solutions in 1% KCl.

**Morphological, biochemical and physiological features**. Common features of the putative *Xanthomonas campestris* and the false isolates are shown in Table 3. In addition to these features, hydrolysis of Tween 80, H S production, mucoid growth on 5% glucose, esculin hydrolysis and growth at 37 and 40°C were unable to differentiate the putative *X. campestris* isolates from most of false isolates (Table 4). All of the false isolates lacked urease and amylase activities, did not produce acid from sucrose, and did not form mucoid colonies on 5% sucrose-containing medium. Most of them did not grow in medium containing 4% NaCl, were nonmotile, and utilized asparagine as sole carbon and nitrogen source. Putative *X. campestris* isolates could grow in presence of 4% NaCl, but none of the false isolates could even grow in presence of 3% NaCl. These biochemical features, shaded in Table 4, differentiated the false isolates from the putative *X. campestris* isolates. Result of biochemical tests for the putative *X. campestris* isolates was in correlation with biochemical features assigned to the genus *Xanthomonas* (11) and *X. campestris* phenon as described by Van den Mooter and Swings (34) and only minor differences were observed among the putative *X. campestris* isolates.

**Table 3:** Common features of the mucoid and yellow-pigmented bacteria isolated on SX agar medium.

Characteristics	Result
Cell morphology	Rods or coccobacilli
Cell arrangement	Single, pair
Gram reaction	−
KOH reaction	+
Catalase	+
Oxidation-Fermentation test	Oxidative
Indole production	+
Acetoin production	−
Arginine dihydrolase	−
Fluorescence on King B	−
Growth at 4C	−
Growth at 36C	+
Growth at initial pH 4.5	−
Growth in 1% NaCl	+

**Table 4:** Biochemical tests differentiating the putative *Xanthomonas campestris* from the false isolates*

Bacteria	Characteristics	Urease	Acid from sucrose	Mucoid growth on 5% sucrose	Hydrolysis of starch	†Growth in 4% NaCl	Motility	Growth in asparagine medium	Utilization of citrate	Utilization of propionate	Growth in 0.1% TTC	Oxidase	Hydrolysis of gelatin	Hydrolysis of casein	Growth at 39°C	Nitrate reduction	Growth in 2% NaCl	Growth at 38°C	Acid from glucose	Growth at 37°C	Growth at 40°C	Hydrolysis of esculin	Mucoid growth on 5% glucose	H_2_S production	Growth in 5% NaCl	Hydrolysis of Tween 80
**Putative*****X. campestris***	**+**	0	100	100	100	100	100	0	100	100	0	0	100	100	7	0	100	93	100	100	0	100	100	100	10	100
	**−**	100	0	0	0	0	0	100	0	0	100	100	0	0	93	100	0	7	0	0	100	0	0	0	90	0

**False isolates**	**+**	100	0	0	0	7	15	78	26	37	59	52	48	52	48	44	56	63	67	70	15	89	93	93	4	96
	**−**	0	100	100	100	93	85	22	74	63	41	48	52	48	52	56	44	37	33	30	85	11	7	7	96	4

***X. campestris*****b82*****X. campestris*****DSM 1706**		**−**	+	+	+	+	+	**−**	+	+	**−**	**−**	+	+	**−**	**−**	+	**−**	+	+	-	+	+	+	**−**	+

* Distinctive biochemical tests, shown in gray shade, are suggested for the differentiation of *X. campestris* from other isolates.

† The same results were obtained using 3% NaCl.

**Fig. 2:**
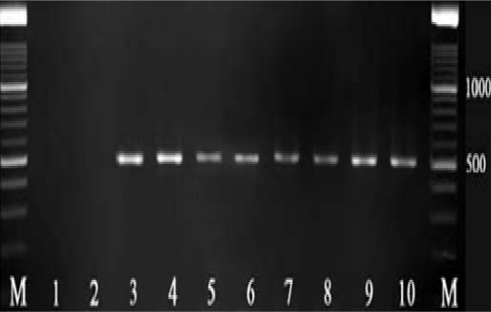
Gel electrophoretic analysis of PCR products amplified from the genomic DNA of the representatives putative *Xanthomonas campestris* isolates using primers HrcCF2 and HrcCR2. Lane M: size marker (DNA Ladder 100 bp plus, Fermentas); Lane 1: SAM 0302; Lane 2: SAM 0401; Lane 3: SAM 3301; Lane 4: SAM 4101; Lane 5: SAM 4204; Lane 6: SAM 4205; Lane 7: SAM 4210; Lane 8: SAM 4213; Lane 9: SAM 4217; Lane10: *X. campestris* DSM 1706 (positive control); Negative controls are not shown.

**Molecular identification**. To confirm the isolation of the *Xanthomonas campestris* from soil of cabbage farms, and to evaluate the biochemical tests carried out in this study, a *X. campestris*-specific gene was targeted for PCR-based identification of nine representative isolates. Desired PCR product with approximately 520 bp was obtained from DNA of seven isolates including SAM 3301, SAM 4101, SAM 4204, SAM 4205, SAM 4210, SAM 4213, SAM 4217, and *X. campestris* DSM 1706 as positive control (Fig. 2). Thus, identification of the isolates as *X. campestris* was confirmed, but in case of the isolates SAM 0302 and SAM 0401, this fragment was not detected.

## DISCUSSION

In this study, the SX agar semi-selective medium was used for isolation of *X. campestris* strains from soil (16). Morphology of colonies and apparent starch hydrolysis are not sufficient parameters of the screening procedure. In addition, there are other bacteria with *Xanthomonas*-like morphology on the SX agar; many of them were discovered when colonies transferred to YM agar and grew without any sign of pigmentation and mucoidity, which usually indicates exopolysaccharide production (37).

We performed this study to facilitate differentiation of *Xanthomonas campestris* from other yellow-pigmented bacteria isolated from soil. Among a great number of biochemical and morphological tests available in the literature (31), a minimum number of tests required as complement of screening procedure is hereby introduced. Most of these tests were selected based on their ability to produce 100% positive or negative results for putative *X. campestris*, in contrast to nearly no results of the same tests obtained for other false isolates. According to these results, we suggest urease activity, acid production from sucrose, mucoid growth on medium containing 5% sucrose, amylase activity, growth in 4% NaCl, motility and utilization of asparagine as sole carbon and nitrogen source for differentiation of *X. campestris* from other mucoid yellow-pigmented bacteria isolated from soil. The outcome of these tests was completely distinctive and could differentiate predominantly *X. campestris* from false yellow-pigmented bacteria isolated from soil. The final validation of the above tests was carried out by the production of xanthomonadin, xanthan exopolysaccharide and tracking of *hrc* gene marker.

One other method to distinguish *Xanthomonas* spp. is to identify the yellow xanthomonadin pigment of *Xanthomonas*, because the pigment is unique to the genus (31, 27). In most members of *Xanthomonas* spp., the methanol extract has a major absorption maximum at 445 (27) or 441 (31) nm, usually with shoulders at wavelengths about 22 nm lower and 25 nm higher; pigments with similar spectral absorption properties are rare or nonexistent in other bacteria (27). Based on the results of absorption spectra for the isolates, this test may be considered as a key test for differentiation of the putative *Xanthomonas campestris* from other mucoid and yellow-pigmented bacteria obtained by the screening procedure, though it can not confirm the presence of xanthomonadins in the isolates.

Capability of biopolymer production in the sucrose-containing medium also differentiated the putative *X. campestris* isolates from the false isolates. Amount of biopolymer production and rheological properties of the biopolymer produced by nine representative isolates were equivalent to those of control strains. These yields were similar with the yields reported from commercial strains (38).

Viscometery of fermentation broths have been used widely as indicator of xanthan production, even when it does not reflect the quality of the polymer produced. High broth viscosities may be due to high concentrations of polymer with low quality. Due to these reasons, we chose to measure the apparent viscosity of aqueous solution of purified polymers. On the other hand, viscosimetry of concentrated xanthan solutions is commonly used to characterize reconstituted polymers but it does not give explicit information about shape or size of the polymer molecule (3).

In this study, presence of a species-specific gene, *hrcC*, was confirmed in seven representative isolates but not for two other isolates. Presence of *hrp* genes is essential for pathogenicity in *Xanthomonas campestris* (26); hence, the positive isolates have at least one of the factors required for pathogenicity. Two negative isolates are not able to cause disease in the family *Brassicaceae*. The isolates may belong to other species or may be considered as opportunistic xanthomonads. In addition to *X. campestris* pv. *campestris*, other starch-hydrolyzing species and pathovars such as *X. campestris* pv. *begoniae*, *X. campestris* pv. *citri*, *X. campestris* pv. *dieffenbachiae*, *X. campestris* pv. *nigromaculans*, *X. juglandis* pv. *corylina*, *X. hyacinthi*, and *X. pisi* can be isolated on SX agar medium (31). These strains may also produce xanthan. Opportunistic xanthomonads are xanthomonad populations, living in close association with plants but causing no apparent disease symptoms on the host and missing the *hrp* genes typical of pathogenic members of the genus (39).

Using the screening procedure accompanied by complement minimal tests for tentative identification of *Xanthomonas campestris* has several advantages. It accelerates easy selection of novel xanthan-producing strains and better control of diseases by xanthomonads. Wide applications of xanthan gum in different industries are expanding globally and development of new local strains of *X. campestris* is necessary (40). The screening of xanthan-producing bacteria in natural environments is a technique which can lead to the isolation of strains with potentially useful traits (38, 8). These strains may differ from each other in quantity and quality of gum production (7–9, 41, 42), period of fermentation (3), and utilization of cheap substrates (5, 43–45).

Furthermore, black rot of crucifers caused by *X. campestris* is a worldwide problem of economic significance (18). Detection of the bacterium in plant residues and soil has a decisive role in control of related plant diseases and preventing economic losses. The tests suggested here are therefore also valuable as confirmatory tests to complete rapid molecular diagnosis and facilitate detection of the bacteria from soil in epidemiologic studies.
